# Tris[μ-1,2-bis­(diphenyl­phosphino)­ethane]-1:2κ^2^ *P*:*P*′;1:3κ^2^ *P*:*P*′;2:3κ^2^ *P*:*P*′-di-μ-bromido-1:2κ^4^ *Br*:*Br*-bromido-3κ*Br*-tricopper(I) acetone hemisolvate

**DOI:** 10.1107/S1600536808032789

**Published:** 2008-10-15

**Authors:** Wen-Juan Shi

**Affiliations:** a Jiangxi Key Laboratory of Surface Engineering, Jiangxi Science and Technology Normal University, Jiangxi 330013, People’s Republic of China

## Abstract

In the crystal structure of the title compound, [Cu_3_Br_3_(C_26_H_24_P_2_)_3_]·0.5CH_3_COCH_3_, two of the Cu centers are bridged by two bromide anions forming a Cu(μ-Br)_2_Cu core, which is further bridged by a 1,2-bis­(diphenyl­phosphino)­ethane (dppe) ligand. The third Cu center is terminally bound to another bromide ligand and is connected to the other two Cu atoms by bridging dppe ligands, forming a triangular cluster unit. The acetone solvent mol­ecule exhibits twofold disorder about an inversion centre at (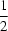, 1, 0). The crystal structure is stabilized by inter­molecular C—H⋯Br hydrogen bonds.

## Related literature

For related structures, see: Albano *et al.* (1972 ▶); Comba *et al.* (1999 ▶); Darensbourg *et al.* (1990 ▶); Effendy *et al.* (2006 ▶); Eller *et al.* (1977 ▶); Leoni *et al.* (1983 ▶); Mohr *et al.* (1991 ▶); Nicola *et al.* (2006 ▶).
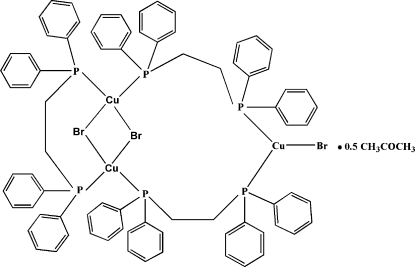


## Experimental

### 

#### Crystal data

[Cu_3_Br_3_(C_26_H_24_P_2_)_3_]·0.5C_3_H_6_O
*M* *_r_* = 1654.56
Monoclinic, 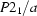

*a* = 18.6878 (10) Å
*b* = 17.1080 (9) Å
*c* = 25.2742 (13) Å
β = 109.924 (1)°
*V* = 7596.8 (7) Å^3^
*Z* = 4
Mo *K*α radiation
μ = 2.58 mm^−1^
*T* = 295 (2) K
0.22 × 0.20 × 0.18 mm

#### Data collection

Bruker SMART APEX area-detector diffractometer
Absorption correction: multi-scan (*SADABS*; Sheldrick, 1996 ▶) *T*_min_ = 0.569, *T*_max_ = 0.625
38020 measured reflections
13376 independent reflections
8616 reflections with *I* > 2σ(*I*)
*R*_int_ = 0.050

#### Refinement

*R*[*F* ^2^ > 2σ(*F* ^2^)] = 0.051
*wR*(*F* ^2^) = 0.140
*S* = 1.03
13376 reflections
715 parameters
29 restraints
H-atom parameters constrained
Δρ_max_ = 0.69 e Å^−3^
Δρ_min_ = −0.57 e Å^−3^

### 

Data collection: *SMART* (Bruker, 2002 ▶); cell refinement: *SAINT* (Bruker, 2002 ▶); data reduction: *SAINT*; program(s) used to solve structure: *SHELXS97* (Sheldrick, 2008 ▶); program(s) used to refine structure: *SHELXL97* (Sheldrick, 2008 ▶); molecular graphics: *SHELXTL* (Sheldrick, 2008 ▶); software used to prepare material for publication: *SHELXTL*.

## Supplementary Material

Crystal structure: contains datablocks I, global. DOI: 10.1107/S1600536808032789/sj2547sup1.cif

Structure factors: contains datablocks I. DOI: 10.1107/S1600536808032789/sj2547Isup2.hkl

Additional supplementary materials:  crystallographic information; 3D view; checkCIF report

## Figures and Tables

**Table 1:** Hydrogen-bond geometry (Å, °)

*D*—H⋯*A*	*D*—H	H⋯*A*	*D*⋯*A*	*D*—H⋯*A*
C24—H24⋯Br1^i^	0.93	2.92	3.560 (3)	127
C65—H65*A*⋯Br1	0.97	2.85	3.576 (5)	132
C40—H40*A*⋯Br2	0.97	2.86	3.675 (5)	142

Symmetry code: (i) 
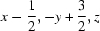
.

**Fig. 1.**
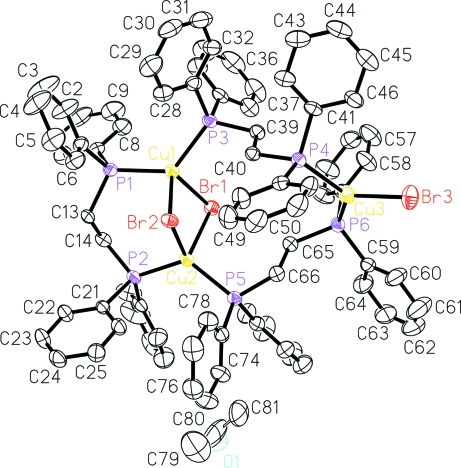
The asymmetric unit of (I), with displacement ellipsoids drawn at the 30% probability level. H atoms are omitted for clarity.

**Fig. 2.**
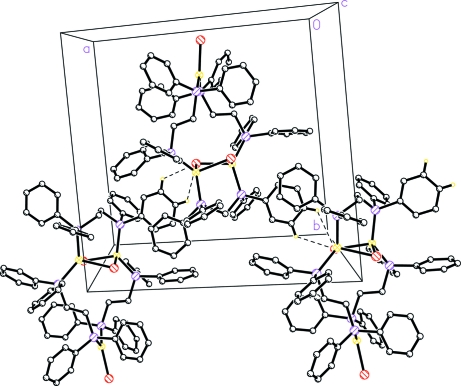
Crystal packing of (I) showing the intermolecular C—H···Br hydrogen bonds as dashed lines.

## supplementary crystallographic information

### Comment

Copper(I) halides react with bidentate phosphine ligands dppe [dppe is 1,2-bis(diphenylphosphino)ethane, C_26_H_24_P_2_] to give a series with the compounds of general formula [Cu_2_*X*_2_(dppe)_3_.solvate] (*X* is a halide anion) (Albano *et al.*, 1972; Comba *et al.*, 1999; Darensbourg *et al.*, 1990; Effendy *et al.*, 2006; Eller *et al.*, 1977; Leoni *et al.*, 1983; Mohr *et al.*, 1991; Nicola *et al.*, 2006). In these compounds Cu centers are bridged by a dppe ligand and each metal center carries one chelating dppe unit, with the fourth coordination site available for the Br^-^ anions. We now report the crystal structure of a triangular cluster (I), Fig 1,  obtained by reaction of CuBr with dppe ligand in acetone as solvent.

The asymmetric unit of the structure consists of a trinuclear molecule Cu_3_Br_3_(dppe)_3_ and half an acetone solvate molecule. In the molecule Cu_3_ Br_3_(dppe)_3_, two copper(I) centers are bridged by the bromide ligands forming a Cu(µ-Br)_2_Cu core, while the third copper(I) is terminally bonded to another bromide ligand. In the dinuclear Cu(µ-Br)_2_Cu core, the Cu(1)···Cu(2) separation is 3.169 (2) Å, while Cu(1)···Cu(3) and Cu(2)···Cu(3) separations are 6.606 (2)Å and 6.537 (1) Å, respectively. The Cu_2_Br_2_ core does not lie in a plane but is bent with a dihedral angle of 27.4 (1) ° between the planes formed by Cu(1), Br(1), Cu(2), and Cu(1), Br(2), Cu2. Each copper cation binds to two bridging dppe ligands forming a triangular tri-copper cluster system. It is interesting that Cu centers have two different coordination environments in this compound, that is CuBr_2_P_2_ in a distorted tetrahedral geometry and CuBrP_2_ in a slightly distorted trigonal planar geometry. In the tetrahedral CuBr_2_P_2_ core, the largest deviation from the ideal geometry is reflected in the P(1)—Cu(1)—P(3) [126.48 (5) °] and P(2)—Cu(2)—P(5) [126.48 (5) °] angles. These values are markedly higher than the normal tetrahedral value of 109.4 °.

The crystal structure is stabilized by intermolecular C—H···Br hydrogen bonds between the Br(1)^-^ anions and –CH groups from phenyl rings, forming a one-dimensional supramolecular array (Fig. 2).

### Experimental

A solution of 1,2-bis(diphenylphosphino)ethane (0.0181 g, 0.05 mmol) was added to a stirred suspension of CuBr (0.0079 g, 0.05 mmol) in acetone (7 ml) and the mixture was stirred and moderately heated. After the formation of a completely clear solution, 5-methoxy-2-benzimidazolethiol (0.0095 g, 0.05 mmol) was added slowly and the stirring was continued for half an hour. The resulting solution was filtered off and stand at room temperature. Colorless crystals were formed after several days. Yield: 7 mg (18%).

### Refinement

All H-atoms were positioned geometrically and refined using a riding model with d(C—H) = 0.93 Å or 0.97 Å, *U*_iso_=1.2*U*_eq_ (C) for aromatic and methylene H atoms; 0.96 Å, *U*_iso_= 1.5*U*_eq_ (C) for CH_3_ groups.  The acetone solvate molecule exhibits 2-fold disorder about an inversion centre at (0.5, 1, 0) and its atoms are refined with occupancy factors of 0.5.

### Crystal data

**Table:**

[Cu_3_Br_3_(C_26_H_24_P_2_)_3_]·0.5C_3_H_6_O	*F*(000) = 3352
*M**_r_* = 1654.56	*D*_x_ = 1.447 Mg m^−^^3^
Monoclinic, *P*2_1_/*a*	Mo *K*α radiation, λ = 0.71073 Å
Hall symbol: -P 2yab	Cell parameters from 4594 reflections
*a* = 18.6878 (10) Å	θ = 2.3–22.5°
*b* = 17.1080 (9) Å	µ = 2.58 mm^−^^1^
*c* = 25.2742 (13) Å	*T* = 295 K
β = 109.924 (1)°	Block, colorless
*V* = 7596.8 (7) Å^3^	0.22 × 0.20 × 0.18 mm
*Z* = 4	

### Data collection

**Table:**

Bruker SMART APEX area-detector diffractometer	13376 independent reflections
Radiation source: fine-focus sealed tube	8616 reflections with *I* > 2σ(*I*)
graphite	*R*_int_ = 0.050
φ and ω scans	θ_max_ = 25.0°, θ_min_ = 1.5°
Absorption correction: multi-scan (SADABS; Sheldrick, 1996)	*h* = −22→16
*T*_min_ = 0.569, *T*_max_ = 0.625	*k* = −20→20
38020 measured reflections	*l* = −30→30

### Refinement

**Table:**

Refinement on *F*^2^	Primary atom site location: structure-invariant direct methods
Least-squares matrix: full	Secondary atom site location: difference Fourier map
*R*[*F*^2^ > 2σ(*F*^2^)] = 0.051	Hydrogen site location: inferred from neighbouring sites
*wR*(*F*^2^) = 0.140	H-atom parameters constrained
*S* = 1.03	*w* = 1/[σ^2^(*F*_o_^2^) + (0.0665*P*)^2^] where *P* = (*F*_o_^2^ + 2*F*_c_^2^)/3
13376 reflections	(Δ/σ)_max_ = 0.001
715 parameters	Δρ_max_ = 0.69 e Å^−^^3^
29 restraints	Δρ_min_ = −0.57 e Å^−^^3^

### Special details

**Table:**

Geometry. All e.s.d.'s (except the e.s.d. in the dihedral angle between two l.s. planes) are estimated using the full covariance matrix. The cell e.s.d.'s are taken into account individually in the estimation of e.s.d.'s in distances, angles and torsion angles; correlations between e.s.d.'s in cell parameters are only used when they are defined by crystal symmetry. An approximate (isotropic) treatment of cell e.s.d.'s is used for estimating e.s.d.'s involving l.s. planes.
Refinement. Refinement of *F*^2^ against ALL reflections. The weighted *R*-factor *wR* and goodness of fit *S* are based on *F*^2^, conventional *R*-factors *R* are based on *F*, with *F* set to zero for negative *F*^2^. The threshold expression of *F*^2^ > σ(*F*^2^) is used only for calculating *R*-factors(gt) *etc*. and is not relevant to the choice of reflections for refinement. *R*-factors based on *F*^2^ are statistically about twice as large as those based on *F*, and *R*- factors based on ALL data will be even larger.

### Fractional atomic coordinates and isotropic or equivalent isotropic displacement parameters (Å^2^)

**Table:**

	*x*	*y*	*z*	*U*_iso_*/*U*_eq_	Occ. (<1)
Br1	0.55058 (3)	0.56272 (3)	0.69646 (2)	0.04792 (16)	
Br2	0.41451 (3)	0.54440 (3)	0.78244 (2)	0.04919 (16)	
Br3	0.48724 (5)	0.07267 (4)	0.73962 (3)	0.0786 (2)	
Cu1	0.55200 (4)	0.58726 (4)	0.79497 (3)	0.04737 (19)	
Cu2	0.41075 (4)	0.57525 (3)	0.68183 (3)	0.04719 (19)	
Cu3	0.50461 (4)	0.20927 (4)	0.74107 (3)	0.04982 (19)	
P1	0.54401 (8)	0.71311 (8)	0.81736 (6)	0.0436 (3)	
P2	0.38504 (8)	0.70347 (8)	0.66503 (6)	0.0446 (3)	
P3	0.64042 (8)	0.50050 (8)	0.84372 (6)	0.0437 (3)	
P4	0.51958 (8)	0.27137 (8)	0.82242 (6)	0.0421 (3)	
P5	0.34513 (8)	0.47459 (8)	0.63000 (6)	0.0455 (4)	
P6	0.50801 (9)	0.27597 (8)	0.66601 (6)	0.0471 (4)	
C1	0.5387 (2)	0.7255 (3)	0.88786 (13)	0.0616 (15)	
C2	0.5996 (2)	0.7566 (3)	0.93130 (17)	0.0910 (17)	
H2	0.6425	0.7744	0.9242	0.109*	
C3	0.5965 (2)	0.7611 (3)	0.98538 (15)	0.112 (2)	
H3	0.6372	0.7819	1.0144	0.135*	
C4	0.5324 (3)	0.7345 (3)	0.99601 (14)	0.113 (2)	
H4	0.5303	0.7375	1.0322	0.136*	
C5	0.4715 (2)	0.7034 (3)	0.95257 (19)	0.093 (2)	
H5	0.4286	0.6856	0.9597	0.111*	
C6	0.4746 (2)	0.6989 (2)	0.89850 (16)	0.0715 (18)	
H6	0.4339	0.6781	0.8694	0.086*	
C7	0.61863 (19)	0.7810 (2)	0.81621 (17)	0.0513 (13)	
C8	0.6873 (2)	0.7492 (2)	0.81675 (18)	0.0713 (15)	
H8	0.6935	0.6952	0.8172	0.086*	
C9	0.74670 (18)	0.7980 (3)	0.81660 (19)	0.0900 (19)	
H9	0.7926	0.7766	0.8170	0.108*	
C10	0.7375 (3)	0.8786 (3)	0.81591 (18)	0.099 (3)	
H10	0.7772	0.9112	0.8158	0.119*	
C11	0.6688 (3)	0.91048 (19)	0.81537 (19)	0.094 (2)	
H11	0.6626	0.9644	0.8149	0.112*	
C12	0.6094 (2)	0.8617 (2)	0.81552 (18)	0.077 (2)	
H12	0.5634	0.8830	0.8152	0.092*	
C13	0.4555 (3)	0.7617 (3)	0.7733 (2)	0.0490 (14)	
H13A	0.4120	0.7310	0.7738	0.059*	
H13B	0.4520	0.8127	0.7890	0.059*	
C14	0.4530 (3)	0.7714 (3)	0.7123 (2)	0.0537 (15)	
H14A	0.5033	0.7619	0.7105	0.064*	
H14B	0.4386	0.8246	0.7000	0.064*	
C15	0.3770 (3)	0.7384 (3)	0.59432 (14)	0.0662 (16)	
C16	0.4259 (2)	0.7941 (3)	0.5855 (2)	0.0971 (19)	
H16	0.4633	0.8166	0.6160	0.116*	
C17	0.4189 (3)	0.8162 (3)	0.5310 (2)	0.113 (2)	
H17	0.4516	0.8535	0.5251	0.135*	
C18	0.3629 (4)	0.7826 (3)	0.48539 (16)	0.112 (3)	
H18	0.3582	0.7974	0.4490	0.135*	
C19	0.3140 (3)	0.7269 (3)	0.49421 (15)	0.116 (3)	
H19	0.2766	0.7044	0.4637	0.139*	
C20	0.3211 (3)	0.7047 (2)	0.5487 (2)	0.091 (2)	
H20	0.2884	0.6675	0.5546	0.109*	
C21	0.29389 (16)	0.7331 (2)	0.67018 (15)	0.0451 (13)	
C22	0.27554 (19)	0.81068 (17)	0.67562 (16)	0.0577 (15)	
H22	0.3113	0.8498	0.6786	0.069*	
C23	0.2037 (2)	0.82991 (16)	0.67656 (17)	0.0670 (17)	
H23	0.1914	0.8818	0.6802	0.080*	
C24	0.15025 (16)	0.7715 (2)	0.67207 (17)	0.0652 (17)	
H24	0.1022	0.7844	0.6727	0.078*	
C25	0.16860 (19)	0.6939 (2)	0.66663 (17)	0.0696 (18)	
H25	0.1328	0.6548	0.6636	0.084*	
C26	0.2404 (2)	0.67467 (15)	0.66568 (16)	0.0599 (16)	
H26	0.2527	0.6227	0.6620	0.072*	
C27	0.6597 (2)	0.4920 (2)	0.91964 (12)	0.0501 (14)	
C28	0.60622 (19)	0.5235 (2)	0.94068 (17)	0.0642 (17)	
H28	0.5653	0.5519	0.9172	0.077*	
C29	0.6140 (3)	0.5124 (3)	0.99685 (19)	0.090 (2)	
H29	0.5782	0.5335	1.0109	0.108*	
C30	0.6751 (3)	0.4699 (3)	1.03198 (12)	0.091 (3)	
H30	0.6803	0.4625	1.0696	0.110*	
C31	0.7286 (2)	0.4384 (2)	1.01093 (17)	0.092 (3)	
H31	0.7695	0.4100	1.0344	0.110*	
C32	0.7208 (2)	0.4495 (2)	0.95476 (19)	0.0667 (18)	
H32	0.7566	0.4284	0.9407	0.080*	
C33	0.73388 (18)	0.5171 (2)	0.83686 (18)	0.0540 (15)	
C34	0.7868 (3)	0.5656 (2)	0.87456 (17)	0.082 (2)	
H34	0.7763	0.5871	0.9049	0.098*	
C35	0.8555 (2)	0.5819 (2)	0.8669 (2)	0.105 (3)	
H35	0.8910	0.6143	0.8921	0.126*	
C36	0.8713 (2)	0.5497 (3)	0.8216 (3)	0.105 (3)	
H36	0.9172	0.5606	0.8165	0.126*	
C37	0.8183 (3)	0.5013 (3)	0.78388 (19)	0.096 (3)	
H37	0.8288	0.4798	0.7535	0.115*	
C38	0.7496 (2)	0.4850 (2)	0.79152 (16)	0.0681 (18)	
H38	0.7142	0.4526	0.7663	0.082*	
C39	0.6189 (3)	0.3980 (3)	0.8223 (2)	0.0452 (13)	
H39A	0.6157	0.3915	0.7834	0.054*	
H39B	0.6590	0.3644	0.8456	0.054*	
C40	0.5426 (3)	0.3758 (3)	0.8288 (2)	0.0422 (13)	
H40A	0.5022	0.4038	0.8006	0.051*	
H40B	0.5431	0.3936	0.8654	0.051*	
C41	0.59175 (19)	0.2275 (2)	0.88371 (14)	0.0481 (14)	
C42	0.6206 (2)	0.26543 (19)	0.93530 (16)	0.0654 (17)	
H42	0.6024	0.3146	0.9400	0.079*	
C43	0.6766 (2)	0.2299 (3)	0.97987 (13)	0.086 (2)	
H43	0.6959	0.2552	1.0144	0.103*	
C44	0.7038 (2)	0.1564 (3)	0.97285 (17)	0.089 (2)	
H44	0.7413	0.1326	1.0027	0.107*	
C45	0.6750 (3)	0.1185 (2)	0.9213 (2)	0.095 (2)	
H45	0.6932	0.0693	0.9166	0.114*	
C46	0.6190 (2)	0.1540 (2)	0.87669 (15)	0.0705 (18)	
H46	0.5997	0.1287	0.8422	0.085*	
C47	0.43185 (17)	0.2678 (2)	0.83960 (15)	0.0470 (13)	
C48	0.3915 (2)	0.33457 (17)	0.84338 (18)	0.0682 (18)	
H48	0.4092	0.3836	0.8377	0.082*	
C49	0.3246 (2)	0.3281 (2)	0.8556 (2)	0.084 (2)	
H49	0.2976	0.3728	0.8581	0.101*	
C50	0.29816 (19)	0.2549 (3)	0.86409 (19)	0.084 (2)	
H50	0.2534	0.2505	0.8723	0.101*	
C51	0.3385 (2)	0.1881 (2)	0.86031 (18)	0.0749 (19)	
H51	0.3208	0.1391	0.8660	0.090*	
C52	0.4054 (2)	0.19459 (17)	0.84807 (16)	0.0584 (16)	
H52	0.4324	0.1499	0.8455	0.070*	
C53	0.60662 (17)	0.2952 (2)	0.67069 (17)	0.0560 (14)	
C54	0.6285 (2)	0.3606 (2)	0.64729 (18)	0.085 (2)	
H54	0.5923	0.3969	0.6275	0.102*	
C55	0.7046 (3)	0.3716 (2)	0.6534 (2)	0.097 (2)	
H55	0.7193	0.4153	0.6378	0.116*	
C56	0.75884 (18)	0.3172 (3)	0.6830 (2)	0.0881 (19)	
H56	0.8098	0.3246	0.6871	0.106*	
C57	0.7369 (2)	0.2519 (2)	0.70636 (19)	0.0943 (18)	
H57	0.7732	0.2155	0.7261	0.113*	
C58	0.6608 (2)	0.2409 (2)	0.70022 (18)	0.0813 (16)	
H58	0.6462	0.1971	0.7159	0.098*	
C59	0.4608 (2)	0.2340 (2)	0.59617 (14)	0.0631 (16)	
C60	0.4242 (3)	0.1626 (3)	0.59348 (17)	0.0912 (19)	
H60	0.4258	0.1373	0.6264	0.109*	
C61	0.3853 (3)	0.1290 (2)	0.5415 (2)	0.116 (2)	
H61	0.3609	0.0812	0.5397	0.139*	
C62	0.3830 (3)	0.1668 (3)	0.49229 (16)	0.120 (3)	
H62	0.3570	0.1443	0.4575	0.144*	
C63	0.4196 (3)	0.2382 (3)	0.49499 (14)	0.106 (3)	
H63	0.4180	0.2635	0.4620	0.127*	
C64	0.4584 (3)	0.2718 (2)	0.54693 (19)	0.086 (2)	
H64	0.4829	0.3196	0.5487	0.104*	
C65	0.4670 (3)	0.3739 (3)	0.6582 (2)	0.0485 (14)	
H65A	0.4972	0.4055	0.6898	0.058*	
H65B	0.4707	0.3971	0.6243	0.058*	
C66	0.3841 (3)	0.3771 (3)	0.6551 (2)	0.0486 (14)	
H66A	0.3808	0.3673	0.6920	0.058*	
H66B	0.3549	0.3372	0.6295	0.058*	
C67	0.3434 (2)	0.4706 (2)	0.55747 (13)	0.0544 (15)	
C68	0.3903 (2)	0.5223 (2)	0.54227 (17)	0.079 (2)	
H68	0.4177	0.5600	0.5677	0.095*	
C69	0.3964 (3)	0.5179 (3)	0.4891 (2)	0.109 (3)	
H69	0.4279	0.5525	0.4789	0.130*	
C70	0.3556 (3)	0.4616 (3)	0.45105 (14)	0.113 (3)	
H70	0.3597	0.4586	0.4154	0.135*	
C71	0.3086 (3)	0.4098 (3)	0.46625 (16)	0.101 (3)	
H71	0.2813	0.3722	0.4408	0.121*	
C72	0.3025 (2)	0.4143 (2)	0.51946 (19)	0.0725 (19)	
H72	0.2711	0.3797	0.5296	0.087*	
C73	0.2464 (3)	0.4682 (3)	0.6269 (3)	0.0545 (15)	
C74	0.1840 (4)	0.4800 (4)	0.5787 (3)	0.084 (2)	
H74	0.1906	0.4875	0.5442	0.101*	
C75	0.1099 (4)	0.4803 (5)	0.5824 (4)	0.101 (2)	
H75	0.0680	0.4871	0.5498	0.121*	
C76	0.0989 (5)	0.4715 (4)	0.6308 (4)	0.096 (2)	
H76	0.0497	0.4721	0.6320	0.116*	
C77	0.1591 (4)	0.4614 (4)	0.6789 (4)	0.092 (2)	
H77	0.1509	0.4563	0.7131	0.110*	
C78	0.2337 (4)	0.4585 (4)	0.6777 (3)	0.0746 (19)	
H78	0.2744	0.4502	0.7108	0.089*	
O1	0.5092 (15)	1.0605 (12)	−0.0348 (9)	0.224 (8)	0.50
C79	0.447 (2)	1.018 (2)	0.0349 (17)	0.255 (14)	0.50
H79A	0.4104	1.0584	0.0207	0.382*	0.50
H79B	0.4741	1.0262	0.0743	0.382*	0.50
H79C	0.4222	0.9680	0.0297	0.382*	0.50
C80	0.5029 (15)	1.0187 (13)	0.0038 (8)	0.256 (11)	0.50
C81	0.5630 (17)	0.9567 (19)	0.0236 (18)	0.254 (13)	0.50
H81A	0.5957	0.9587	0.0014	0.381*	0.50
H81B	0.5392	0.9062	0.0195	0.381*	0.50
H81C	0.5925	0.9654	0.0624	0.381*	0.50

### Atomic displacement parameters (Å^2^)

**Table:**

	*U*^11^	*U*^22^	*U*^33^	*U*^12^	*U*^13^	*U*^23^
Br1	0.0362 (3)	0.0551 (3)	0.0565 (3)	−0.0032 (2)	0.0210 (3)	−0.0022 (3)
Br2	0.0351 (3)	0.0609 (4)	0.0527 (3)	0.0019 (3)	0.0165 (3)	0.0054 (3)
Br3	0.1066 (6)	0.0527 (4)	0.0905 (5)	−0.0089 (4)	0.0519 (5)	−0.0050 (3)
Cu1	0.0386 (4)	0.0478 (4)	0.0532 (4)	0.0049 (3)	0.0125 (3)	−0.0006 (3)
Cu2	0.0392 (4)	0.0463 (4)	0.0537 (4)	0.0049 (3)	0.0126 (3)	−0.0005 (3)
Cu3	0.0511 (4)	0.0495 (4)	0.0530 (4)	0.0031 (3)	0.0230 (4)	−0.0004 (3)
P1	0.0336 (8)	0.0491 (8)	0.0471 (8)	0.0011 (6)	0.0126 (7)	−0.0051 (6)
P2	0.0386 (8)	0.0498 (8)	0.0453 (8)	0.0104 (7)	0.0141 (7)	0.0044 (6)
P3	0.0321 (8)	0.0473 (8)	0.0502 (8)	0.0014 (6)	0.0121 (7)	0.0022 (6)
P4	0.0394 (8)	0.0432 (8)	0.0451 (8)	0.0007 (6)	0.0163 (7)	0.0025 (6)
P5	0.0369 (8)	0.0456 (8)	0.0533 (9)	0.0010 (6)	0.0144 (7)	−0.0009 (6)
P6	0.0505 (9)	0.0472 (8)	0.0443 (8)	0.0070 (7)	0.0170 (7)	−0.0016 (6)
C1	0.057 (3)	0.087 (4)	0.046 (3)	−0.016 (3)	0.024 (3)	−0.017 (3)
C2	0.084 (4)	0.136 (4)	0.056 (3)	−0.040 (3)	0.028 (3)	−0.022 (3)
C3	0.106 (4)	0.167 (5)	0.064 (3)	−0.052 (4)	0.029 (3)	−0.030 (3)
C4	0.113 (5)	0.173 (5)	0.061 (4)	−0.052 (5)	0.038 (4)	−0.032 (4)
C5	0.080 (6)	0.131 (6)	0.083 (5)	−0.019 (5)	0.048 (5)	−0.014 (5)
C6	0.059 (4)	0.101 (5)	0.061 (4)	−0.008 (4)	0.028 (4)	−0.017 (3)
C7	0.043 (3)	0.061 (3)	0.049 (3)	−0.010 (3)	0.015 (3)	−0.005 (2)
C8	0.050 (3)	0.089 (4)	0.076 (3)	−0.013 (3)	0.024 (3)	−0.007 (3)
C9	0.057 (4)	0.117 (5)	0.098 (4)	−0.017 (3)	0.028 (3)	−0.011 (4)
C10	0.091 (6)	0.138 (7)	0.072 (5)	−0.070 (6)	0.032 (5)	−0.024 (5)
C11	0.110 (7)	0.074 (5)	0.100 (6)	−0.030 (5)	0.040 (5)	−0.002 (4)
C12	0.064 (5)	0.065 (4)	0.104 (5)	−0.017 (4)	0.031 (4)	−0.009 (4)
C13	0.032 (3)	0.053 (3)	0.059 (3)	0.010 (2)	0.012 (3)	−0.007 (3)
C14	0.045 (3)	0.058 (4)	0.057 (4)	0.008 (3)	0.015 (3)	0.009 (3)
C15	0.060 (4)	0.080 (4)	0.068 (3)	0.031 (3)	0.034 (3)	0.023 (3)
C16	0.082 (4)	0.125 (4)	0.089 (4)	0.015 (3)	0.034 (3)	0.037 (3)
C17	0.097 (5)	0.149 (5)	0.100 (4)	0.010 (4)	0.043 (4)	0.049 (4)
C18	0.141 (9)	0.138 (8)	0.085 (6)	0.066 (6)	0.073 (6)	0.040 (5)
C19	0.164 (10)	0.117 (7)	0.052 (5)	0.036 (6)	0.019 (5)	0.009 (4)
C20	0.121 (7)	0.093 (5)	0.045 (4)	0.013 (5)	0.011 (4)	0.005 (4)
C21	0.043 (3)	0.053 (3)	0.039 (3)	0.011 (3)	0.013 (3)	−0.001 (2)
C22	0.053 (4)	0.050 (3)	0.073 (4)	0.010 (3)	0.025 (3)	0.004 (3)
C23	0.064 (4)	0.059 (4)	0.089 (5)	0.016 (3)	0.041 (4)	0.006 (3)
C24	0.048 (4)	0.081 (5)	0.074 (4)	0.015 (3)	0.030 (3)	−0.001 (3)
C25	0.050 (4)	0.068 (4)	0.096 (5)	−0.010 (3)	0.031 (4)	−0.015 (4)
C26	0.045 (4)	0.058 (4)	0.080 (4)	0.006 (3)	0.025 (3)	−0.010 (3)
C27	0.042 (3)	0.050 (3)	0.052 (3)	−0.009 (3)	0.009 (3)	−0.003 (3)
C28	0.060 (4)	0.076 (4)	0.053 (4)	−0.007 (3)	0.015 (3)	0.004 (3)
C29	0.091 (6)	0.116 (6)	0.071 (5)	−0.019 (5)	0.039 (5)	−0.014 (4)
C30	0.117 (7)	0.089 (5)	0.050 (4)	−0.032 (5)	0.005 (5)	0.002 (4)
C31	0.092 (6)	0.082 (5)	0.069 (5)	−0.012 (4)	−0.013 (5)	0.008 (4)
C32	0.062 (4)	0.065 (4)	0.060 (4)	0.002 (3)	0.003 (4)	0.004 (3)
C33	0.035 (3)	0.047 (3)	0.076 (4)	0.008 (3)	0.014 (3)	0.015 (3)
C34	0.046 (4)	0.083 (5)	0.111 (6)	−0.013 (4)	0.020 (4)	−0.007 (4)
C35	0.049 (5)	0.102 (6)	0.159 (9)	−0.024 (4)	0.028 (5)	0.005 (5)
C36	0.051 (5)	0.101 (6)	0.175 (9)	0.001 (4)	0.055 (6)	0.047 (6)
C37	0.079 (6)	0.092 (6)	0.144 (7)	0.010 (4)	0.074 (6)	0.031 (5)
C38	0.055 (4)	0.067 (4)	0.092 (5)	−0.001 (3)	0.037 (4)	0.016 (4)
C39	0.038 (3)	0.049 (3)	0.052 (3)	0.002 (2)	0.019 (3)	0.003 (2)
C40	0.037 (3)	0.044 (3)	0.045 (3)	−0.002 (2)	0.013 (3)	0.002 (2)
C41	0.031 (3)	0.053 (3)	0.057 (4)	0.002 (2)	0.011 (3)	0.017 (3)
C42	0.056 (4)	0.080 (4)	0.056 (4)	0.007 (3)	0.013 (3)	0.015 (3)
C43	0.077 (5)	0.115 (6)	0.055 (4)	0.001 (4)	0.009 (4)	0.015 (4)
C44	0.070 (5)	0.101 (6)	0.084 (6)	0.015 (4)	0.011 (5)	0.043 (5)
C45	0.091 (6)	0.082 (5)	0.109 (6)	0.033 (5)	0.029 (5)	0.034 (5)
C46	0.064 (4)	0.062 (4)	0.075 (4)	0.017 (3)	0.011 (4)	0.014 (3)
C47	0.039 (3)	0.061 (3)	0.040 (3)	−0.003 (3)	0.011 (3)	−0.002 (3)
C48	0.049 (4)	0.064 (4)	0.103 (5)	−0.003 (3)	0.040 (4)	−0.003 (4)
C49	0.060 (5)	0.076 (5)	0.129 (6)	−0.001 (4)	0.047 (5)	−0.019 (4)
C50	0.059 (5)	0.105 (6)	0.100 (5)	−0.033 (4)	0.043 (4)	−0.032 (5)
C51	0.068 (5)	0.076 (5)	0.091 (5)	−0.028 (4)	0.041 (4)	−0.013 (4)
C52	0.057 (4)	0.055 (4)	0.072 (4)	−0.019 (3)	0.033 (3)	−0.010 (3)
C53	0.044 (3)	0.068 (3)	0.059 (3)	0.011 (3)	0.021 (3)	0.001 (3)
C54	0.052 (4)	0.097 (5)	0.107 (6)	0.010 (4)	0.031 (4)	0.040 (4)
C55	0.071 (5)	0.106 (6)	0.118 (6)	0.002 (5)	0.040 (5)	0.029 (5)
C56	0.056 (4)	0.108 (5)	0.103 (4)	0.007 (4)	0.029 (3)	−0.007 (4)
C57	0.062 (3)	0.101 (4)	0.114 (4)	0.016 (3)	0.022 (3)	0.008 (3)
C58	0.057 (3)	0.085 (3)	0.098 (4)	0.014 (3)	0.021 (3)	0.012 (3)
C59	0.058 (4)	0.072 (4)	0.052 (3)	0.008 (3)	0.010 (3)	−0.013 (3)
C60	0.089 (4)	0.096 (4)	0.069 (3)	−0.012 (3)	0.001 (3)	−0.016 (3)
C61	0.114 (5)	0.116 (5)	0.089 (4)	−0.022 (4)	−0.003 (4)	−0.022 (4)
C62	0.115 (8)	0.144 (8)	0.073 (6)	0.007 (6)	−0.005 (5)	−0.027 (6)
C63	0.129 (8)	0.126 (7)	0.046 (4)	0.023 (6)	0.011 (5)	−0.005 (4)
C64	0.110 (6)	0.093 (5)	0.054 (4)	0.001 (4)	0.025 (4)	−0.005 (4)
C65	0.046 (3)	0.049 (3)	0.051 (3)	0.003 (3)	0.017 (3)	0.001 (2)
C66	0.047 (3)	0.049 (3)	0.053 (3)	−0.001 (3)	0.020 (3)	0.003 (2)
C67	0.054 (4)	0.054 (3)	0.051 (3)	0.008 (3)	0.012 (3)	0.004 (3)
C68	0.113 (6)	0.068 (4)	0.059 (4)	−0.012 (4)	0.033 (4)	0.004 (3)
C69	0.165 (7)	0.105 (6)	0.069 (5)	−0.016 (5)	0.057 (5)	0.012 (4)
C70	0.162 (7)	0.112 (6)	0.061 (4)	0.007 (5)	0.034 (5)	0.003 (4)
C71	0.128 (7)	0.094 (5)	0.066 (5)	0.001 (5)	0.013 (5)	−0.014 (4)
C72	0.074 (5)	0.069 (4)	0.067 (4)	−0.003 (4)	0.014 (4)	−0.008 (3)
C73	0.044 (4)	0.045 (3)	0.075 (4)	0.000 (3)	0.019 (3)	−0.008 (3)
C74	0.047 (4)	0.102 (5)	0.098 (5)	0.014 (4)	0.017 (4)	−0.010 (4)
C75	0.049 (4)	0.115 (5)	0.125 (6)	0.008 (4)	0.013 (4)	−0.014 (5)
C76	0.055 (4)	0.094 (4)	0.140 (6)	−0.007 (4)	0.034 (4)	−0.017 (5)
C77	0.076 (5)	0.089 (5)	0.130 (6)	−0.011 (4)	0.061 (5)	−0.005 (4)
C78	0.043 (4)	0.080 (5)	0.109 (6)	−0.013 (3)	0.036 (4)	−0.010 (4)
O1	0.216 (19)	0.203 (18)	0.246 (19)	−0.043 (15)	0.069 (16)	0.061 (14)
C79	0.23 (3)	0.23 (2)	0.29 (3)	−0.08 (2)	0.07 (2)	0.02 (2)
C80	0.24 (2)	0.212 (19)	0.28 (2)	−0.059 (18)	0.033 (19)	−0.001 (18)
C81	0.25 (3)	0.18 (2)	0.27 (3)	−0.05 (2)	0.00 (2)	−0.03 (2)

### Geometric parameters (Å, °)

**Table:**

Br1—Cu1	2.5162 (9)	C35—C36	1.3900
Br1—Cu2	2.5204 (8)	C35—H35	0.9300
Br2—Cu2	2.5746 (9)	C36—C37	1.3900
Br2—Cu1	2.5855 (9)	C36—H36	0.9300
Br3—Cu3	2.3581 (9)	C37—C38	1.3900
Cu1—P1	2.2440 (15)	C37—H37	0.9300
Cu1—P3	2.2502 (15)	C38—H38	0.9300
Cu2—P2	2.2547 (15)	C39—C40	1.539 (7)
Cu2—P5	2.2561 (15)	C39—H39A	0.9700
Cu3—P6	2.2332 (16)	C39—H39B	0.9700
Cu3—P4	2.2454 (15)	C40—H40A	0.9700
P1—C7	1.823 (3)	C40—H40B	0.9700
P1—C1	1.830 (3)	C41—C42	1.3900
P1—C13	1.847 (5)	C41—C46	1.3900
P2—C21	1.824 (3)	C42—C43	1.3900
P2—C14	1.831 (5)	C42—H42	0.9300
P2—C15	1.841 (3)	C43—C44	1.3900
P3—C27	1.833 (3)	C43—H43	0.9300
P3—C33	1.835 (3)	C44—C45	1.3900
P3—C39	1.838 (5)	C44—H44	0.9300
P4—C40	1.832 (5)	C45—C46	1.3900
P4—C41	1.832 (3)	C45—H45	0.9300
P4—C47	1.835 (3)	C46—H46	0.9300
P5—C73	1.823 (6)	C47—C48	1.3900
P5—C67	1.824 (3)	C47—C52	1.3900
P5—C66	1.845 (5)	C48—C49	1.3900
P6—C65	1.824 (5)	C48—H48	0.9300
P6—C59	1.830 (3)	C49—C50	1.3900
P6—C53	1.836 (3)	C49—H49	0.9300
C1—C2	1.3900	C50—C51	1.3900
C1—C6	1.3900	C50—H50	0.9300
C2—C3	1.3900	C51—C52	1.3900
C2—H2	0.9300	C51—H51	0.9300
C3—C4	1.3900	C52—H52	0.9300
C3—H3	0.9300	C53—C54	1.3900
C4—C5	1.3900	C53—C58	1.3900
C4—H4	0.9300	C54—C55	1.3900
C5—C6	1.3900	C54—H54	0.9300
C5—H5	0.9300	C55—C56	1.3900
C6—H6	0.9300	C55—H55	0.9300
C7—C8	1.3900	C56—C57	1.3900
C7—C12	1.3900	C56—H56	0.9300
C8—C9	1.3900	C57—C58	1.3900
C8—H8	0.9300	C57—H57	0.9300
C9—C10	1.3900	C58—H58	0.9300
C9—H9	0.9300	C59—C60	1.3900
C10—C11	1.3900	C59—C64	1.3900
C10—H10	0.9300	C60—C61	1.3900
C11—C12	1.3900	C60—H60	0.9300
C11—H11	0.9300	C61—C62	1.3900
C12—H12	0.9300	C61—H61	0.9300
C13—C14	1.537 (7)	C62—C63	1.3900
C13—H13A	0.9700	C62—H62	0.9300
C13—H13B	0.9700	C63—C64	1.3900
C14—H14A	0.9700	C63—H63	0.9300
C14—H14B	0.9700	C64—H64	0.9300
C15—C16	1.3900	C65—C66	1.525 (7)
C15—C20	1.3900	C65—H65A	0.9700
C16—C17	1.3900	C65—H65B	0.9700
C16—H16	0.9300	C66—H66A	0.9700
C17—C18	1.3900	C66—H66B	0.9700
C17—H17	0.9300	C67—C68	1.3900
C18—C19	1.3900	C67—C72	1.3900
C18—H18	0.9300	C68—C69	1.3900
C19—C20	1.3900	C68—H68	0.9300
C19—H19	0.9300	C69—C70	1.3900
C20—H20	0.9300	C69—H69	0.9300
C21—C22	1.3900	C70—C71	1.3900
C21—C26	1.3900	C70—H70	0.9300
C22—C23	1.3900	C71—C72	1.3900
C22—H22	0.9300	C71—H71	0.9300
C23—C24	1.3900	C72—H72	0.9300
C23—H23	0.9300	C73—C74	1.385 (8)
C24—C25	1.3900	C73—C78	1.393 (8)
C24—H24	0.9300	C74—C75	1.420 (10)
C25—C26	1.3900	C74—H74	0.9300
C25—H25	0.9300	C75—C76	1.316 (10)
C26—H26	0.9300	C75—H75	0.9300
C27—C28	1.3900	C76—C77	1.358 (10)
C27—C32	1.3900	C76—H76	0.9300
C28—C29	1.3900	C77—C78	1.404 (9)
C28—H28	0.9300	C77—H77	0.9300
C29—C30	1.3900	C78—H78	0.9300
C29—H29	0.9300	O1—C80	1.248 (10)
C30—C31	1.3900	C79—C80	1.500 (10)
C30—H30	0.9300	C79—H79A	0.9600
C31—C32	1.3900	C79—H79B	0.9600
C31—H31	0.9300	C79—H79C	0.9600
C32—H32	0.9300	C80—C81	1.502 (10)
C33—C34	1.3900	C81—H81A	0.9600
C33—C38	1.3900	C81—H81B	0.9600
C34—C35	1.3900	C81—H81C	0.9600
C34—H34	0.9300		
			
Cu1—Br1—Cu2	77.99 (3)	C35—C34—H34	120.0
Cu2—Br2—Cu1	75.78 (3)	C36—C35—C34	120.0
P1—Cu1—P3	126.47 (6)	C36—C35—H35	120.0
P1—Cu1—Br1	115.49 (5)	C34—C35—H35	120.0
P3—Cu1—Br1	100.95 (5)	C37—C36—C35	120.0
P1—Cu1—Br2	98.87 (4)	C37—C36—H36	120.0
P3—Cu1—Br2	113.18 (5)	C35—C36—H36	120.0
Br1—Cu1—Br2	99.00 (3)	C36—C37—C38	120.0
P2—Cu2—P5	126.48 (6)	C36—C37—H37	120.0
P2—Cu2—Br1	104.72 (5)	C38—C37—H37	120.0
P5—Cu2—Br1	110.58 (5)	C37—C38—C33	120.0
P2—Cu2—Br2	108.52 (5)	C37—C38—H38	120.0
P5—Cu2—Br2	104.09 (5)	C33—C38—H38	120.0
Br1—Cu2—Br2	99.19 (3)	C40—C39—P3	108.8 (3)
P6—Cu3—P4	120.35 (6)	C40—C39—H39A	109.9
P6—Cu3—Br3	122.47 (5)	P3—C39—H39A	109.9
P4—Cu3—Br3	117.18 (5)	C40—C39—H39B	109.9
C7—P1—C1	103.71 (19)	P3—C39—H39B	109.9
C7—P1—C13	103.7 (2)	H39A—C39—H39B	108.3
C1—P1—C13	101.5 (2)	C39—C40—P4	115.4 (3)
C7—P1—Cu1	119.38 (14)	C39—C40—H40A	108.4
C1—P1—Cu1	112.77 (15)	P4—C40—H40A	108.4
C13—P1—Cu1	113.77 (17)	C39—C40—H40B	108.4
C21—P2—C14	103.6 (2)	P4—C40—H40B	108.4
C21—P2—C15	102.11 (19)	H40A—C40—H40B	107.5
C14—P2—C15	103.7 (2)	C42—C41—C46	120.0
C21—P2—Cu2	113.64 (13)	C42—C41—P4	122.7 (2)
C14—P2—Cu2	115.99 (18)	C46—C41—P4	117.3 (2)
C15—P2—Cu2	116.10 (15)	C41—C42—C43	120.0
C27—P3—C33	104.93 (19)	C41—C42—H42	120.0
C27—P3—C39	100.2 (2)	C43—C42—H42	120.0
C33—P3—C39	103.9 (2)	C44—C43—C42	120.0
C27—P3—Cu1	118.09 (15)	C44—C43—H43	120.0
C33—P3—Cu1	113.00 (13)	C42—C43—H43	120.0
C39—P3—Cu1	114.92 (17)	C45—C44—C43	120.0
C40—P4—C41	104.0 (2)	C45—C44—H44	120.0
C40—P4—C47	102.3 (2)	C43—C44—H44	120.0
C41—P4—C47	104.24 (18)	C46—C45—C44	120.0
C40—P4—Cu3	119.66 (17)	C46—C45—H45	120.0
C41—P4—Cu3	113.99 (14)	C44—C45—H45	120.0
C47—P4—Cu3	111.01 (13)	C45—C46—C41	120.0
C73—P5—C67	106.5 (2)	C45—C46—H46	120.0
C73—P5—C66	103.6 (2)	C41—C46—H46	120.0
C67—P5—C66	100.4 (2)	C48—C47—C52	120.0
C73—P5—Cu2	114.33 (18)	C48—C47—P4	122.6 (2)
C67—P5—Cu2	115.63 (15)	C52—C47—P4	117.4 (2)
C66—P5—Cu2	114.70 (18)	C47—C48—C49	120.0
C65—P6—C59	102.1 (2)	C47—C48—H48	120.0
C65—P6—C53	102.6 (2)	C49—C48—H48	120.0
C59—P6—C53	106.0 (2)	C50—C49—C48	120.0
C65—P6—Cu3	115.36 (18)	C50—C49—H49	120.0
C59—P6—Cu3	118.36 (16)	C48—C49—H49	120.0
C53—P6—Cu3	110.82 (15)	C49—C50—C51	120.0
C2—C1—C6	120.0	C49—C50—H50	120.0
C2—C1—P1	120.8 (2)	C51—C50—H50	120.0
C6—C1—P1	119.1 (2)	C52—C51—C50	120.0
C3—C2—C1	120.0	C52—C51—H51	120.0
C3—C2—H2	120.0	C50—C51—H51	120.0
C1—C2—H2	120.0	C51—C52—C47	120.0
C4—C3—C2	120.0	C51—C52—H52	120.0
C4—C3—H3	120.0	C47—C52—H52	120.0
C2—C3—H3	120.0	C54—C53—C58	120.0
C3—C4—C5	120.0	C54—C53—P6	123.5 (2)
C3—C4—H4	120.0	C58—C53—P6	116.5 (2)
C5—C4—H4	120.0	C55—C54—C53	120.0
C6—C5—C4	120.0	C55—C54—H54	120.0
C6—C5—H5	120.0	C53—C54—H54	120.0
C4—C5—H5	120.0	C54—C55—C56	120.0
C5—C6—C1	120.0	C54—C55—H55	120.0
C5—C6—H6	120.0	C56—C55—H55	120.0
C1—C6—H6	120.0	C55—C56—C57	120.0
C8—C7—C12	120.0	C55—C56—H56	120.0
C8—C7—P1	117.3 (3)	C57—C56—H56	120.0
C12—C7—P1	122.7 (3)	C58—C57—C56	120.0
C9—C8—C7	120.0	C58—C57—H57	120.0
C9—C8—H8	120.0	C56—C57—H57	120.0
C7—C8—H8	120.0	C57—C58—C53	120.0
C10—C9—C8	120.0	C57—C58—H58	120.0
C10—C9—H9	120.0	C53—C58—H58	120.0
C8—C9—H9	120.0	C60—C59—C64	120.0
C9—C10—C11	120.0	C60—C59—P6	117.4 (3)
C9—C10—H10	120.0	C64—C59—P6	122.6 (3)
C11—C10—H10	120.0	C61—C60—C59	120.0
C12—C11—C10	120.0	C61—C60—H60	120.0
C12—C11—H11	120.0	C59—C60—H60	120.0
C10—C11—H11	120.0	C60—C61—C62	120.0
C11—C12—C7	120.0	C60—C61—H61	120.0
C11—C12—H12	120.0	C62—C61—H61	120.0
C7—C12—H12	120.0	C63—C62—C61	120.0
C14—C13—P1	111.6 (4)	C63—C62—H62	120.0
C14—C13—H13A	109.3	C61—C62—H62	120.0
P1—C13—H13A	109.3	C62—C63—C64	120.0
C14—C13—H13B	109.3	C62—C63—H63	120.0
P1—C13—H13B	109.3	C64—C63—H63	120.0
H13A—C13—H13B	108.0	C63—C64—C59	120.0
C13—C14—P2	111.3 (4)	C63—C64—H64	120.0
C13—C14—H14A	109.4	C59—C64—H64	120.0
P2—C14—H14A	109.4	C66—C65—P6	114.9 (4)
C13—C14—H14B	109.4	C66—C65—H65A	108.6
P2—C14—H14B	109.4	P6—C65—H65A	108.6
H14A—C14—H14B	108.0	C66—C65—H65B	108.6
C16—C15—C20	120.0	P6—C65—H65B	108.6
C16—C15—P2	122.5 (3)	H65A—C65—H65B	107.5
C20—C15—P2	117.5 (3)	C65—C66—P5	109.0 (3)
C15—C16—C17	120.0	C65—C66—H66A	109.9
C15—C16—H16	120.0	P5—C66—H66A	109.9
C17—C16—H16	120.0	C65—C66—H66B	109.9
C18—C17—C16	120.0	P5—C66—H66B	109.9
C18—C17—H17	120.0	H66A—C66—H66B	108.3
C16—C17—H17	120.0	C68—C67—C72	120.0
C17—C18—C19	120.0	C68—C67—P5	117.2 (3)
C17—C18—H18	120.0	C72—C67—P5	122.6 (3)
C19—C18—H18	120.0	C67—C68—C69	120.0
C20—C19—C18	120.0	C67—C68—H68	120.0
C20—C19—H19	120.0	C69—C68—H68	120.0
C18—C19—H19	120.0	C68—C69—C70	120.0
C19—C20—C15	120.0	C68—C69—H69	120.0
C19—C20—H20	120.0	C70—C69—H69	120.0
C15—C20—H20	120.0	C71—C70—C69	120.0
C22—C21—C26	120.0	C71—C70—H70	120.0
C22—C21—P2	122.7 (2)	C69—C70—H70	120.0
C26—C21—P2	117.2 (2)	C70—C71—C72	120.0
C21—C22—C23	120.0	C70—C71—H71	120.0
C21—C22—H22	120.0	C72—C71—H71	120.0
C23—C22—H22	120.0	C71—C72—C67	120.0
C22—C23—C24	120.0	C71—C72—H72	120.0
C22—C23—H23	120.0	C67—C72—H72	120.0
C24—C23—H23	120.0	C74—C73—C78	118.1 (6)
C25—C24—C23	120.0	C74—C73—P5	124.4 (5)
C25—C24—H24	120.0	C78—C73—P5	117.2 (5)
C23—C24—H24	120.0	C73—C74—C75	119.3 (7)
C24—C25—C26	120.0	C73—C74—H74	120.3
C24—C25—H25	120.0	C75—C74—H74	120.3
C26—C25—H25	120.0	C76—C75—C74	121.7 (8)
C25—C26—C21	120.0	C76—C75—H75	119.1
C25—C26—H26	120.0	C74—C75—H75	119.1
C21—C26—H26	120.0	C75—C76—C77	120.2 (8)
C28—C27—C32	120.0	C75—C76—H76	119.9
C28—C27—P3	117.3 (3)	C77—C76—H76	119.9
C32—C27—P3	122.4 (3)	C76—C77—C78	120.7 (8)
C27—C28—C29	120.0	C76—C77—H77	119.7
C27—C28—H28	120.0	C78—C77—H77	119.7
C29—C28—H28	120.0	C73—C78—C77	119.9 (7)
C30—C29—C28	120.0	C73—C78—H78	120.0
C30—C29—H29	120.0	C77—C78—H78	120.0
C28—C29—H29	120.0	C80—C79—H79A	109.5
C29—C30—C31	120.0	C80—C79—H79B	109.5
C29—C30—H30	120.0	H79A—C79—H79B	109.5
C31—C30—H30	120.0	C80—C79—H79C	109.5
C32—C31—C30	120.0	H79A—C79—H79C	109.5
C32—C31—H31	120.0	H79B—C79—H79C	109.5
C30—C31—H31	120.0	O1—C80—C79	133 (3)
C31—C32—C27	120.0	O1—C80—C81	114 (2)
C31—C32—H32	120.0	C79—C80—C81	112.9 (10)
C27—C32—H32	120.0	C80—C81—H81A	109.5
C34—C33—C38	120.0	C80—C81—H81B	109.5
C34—C33—P3	120.4 (3)	H81A—C81—H81B	109.5
C38—C33—P3	119.5 (3)	C80—C81—H81C	109.5
C33—C34—C35	120.0	H81A—C81—H81C	109.5
C33—C34—H34	120.0	H81B—C81—H81C	109.5
			
Cu2—Br1—Cu1—P1	−82.86 (5)	Cu1—P3—C27—C28	16.4 (3)
Cu2—Br1—Cu1—P3	137.42 (4)	C33—P3—C27—C32	−42.8 (3)
Cu2—Br1—Cu1—Br2	21.53 (3)	C39—P3—C27—C32	64.6 (3)
Cu2—Br2—Cu1—P1	96.50 (4)	Cu1—P3—C27—C32	−169.77 (17)
Cu2—Br2—Cu1—P3	−127.34 (5)	C32—C27—C28—C29	0.0
Cu2—Br2—Cu1—Br1	−21.26 (3)	P3—C27—C28—C29	174.0 (3)
Cu1—Br1—Cu2—P2	90.40 (5)	C27—C28—C29—C30	0.0
Cu1—Br1—Cu2—P5	−130.56 (5)	C28—C29—C30—C31	0.0
Cu1—Br1—Cu2—Br2	−21.64 (3)	C29—C30—C31—C32	0.0
Cu1—Br2—Cu2—P2	−87.77 (5)	C30—C31—C32—C27	0.0
Cu1—Br2—Cu2—P5	135.29 (5)	C28—C27—C32—C31	0.0
Cu1—Br2—Cu2—Br1	21.23 (3)	P3—C27—C32—C31	−173.6 (3)
P3—Cu1—P1—C7	65.97 (18)	C27—P3—C33—C34	−39.8 (3)
Br1—Cu1—P1—C7	−61.92 (17)	C39—P3—C33—C34	−144.6 (3)
Br2—Cu1—P1—C7	−166.39 (16)	Cu1—P3—C33—C34	90.2 (2)
P3—Cu1—P1—C1	−56.12 (17)	C27—P3—C33—C38	144.3 (2)
Br1—Cu1—P1—C1	176.00 (15)	C39—P3—C33—C38	39.5 (3)
Br2—Cu1—P1—C1	71.53 (16)	Cu1—P3—C33—C38	−85.7 (2)
P3—Cu1—P1—C13	−171.1 (2)	C38—C33—C34—C35	0.0
Br1—Cu1—P1—C13	61.1 (2)	P3—C33—C34—C35	−175.8 (3)
Br2—Cu1—P1—C13	−43.4 (2)	C33—C34—C35—C36	0.0
P5—Cu2—P2—C21	64.16 (16)	C34—C35—C36—C37	0.0
Br1—Cu2—P2—C21	−165.59 (14)	C35—C36—C37—C38	0.0
Br2—Cu2—P2—C21	−60.39 (14)	C36—C37—C38—C33	0.0
P5—Cu2—P2—C14	−176.0 (2)	C34—C33—C38—C37	0.0
Br1—Cu2—P2—C14	−45.7 (2)	P3—C33—C38—C37	175.9 (3)
Br2—Cu2—P2—C14	59.5 (2)	C27—P3—C39—C40	69.2 (4)
P5—Cu2—P2—C15	−53.84 (19)	C33—P3—C39—C40	177.5 (3)
Br1—Cu2—P2—C15	76.42 (18)	Cu1—P3—C39—C40	−58.5 (4)
Br2—Cu2—P2—C15	−178.39 (17)	P3—C39—C40—P4	−168.9 (3)
P1—Cu1—P3—C27	49.44 (17)	C41—P4—C40—C39	66.2 (4)
Br1—Cu1—P3—C27	−177.08 (15)	C47—P4—C40—C39	174.5 (4)
Br2—Cu1—P3—C27	−72.24 (15)	Cu3—P4—C40—C39	−62.4 (4)
P1—Cu1—P3—C33	−73.51 (18)	C40—P4—C41—C42	34.5 (3)
Br1—Cu1—P3—C33	59.97 (16)	C47—P4—C41—C42	−72.3 (3)
Br2—Cu1—P3—C33	164.81 (16)	Cu3—P4—C41—C42	166.5 (2)
P1—Cu1—P3—C39	167.50 (19)	C40—P4—C41—C46	−144.0 (3)
Br1—Cu1—P3—C39	−59.02 (19)	C47—P4—C41—C46	109.2 (2)
Br2—Cu1—P3—C39	45.8 (2)	Cu3—P4—C41—C46	−12.0 (2)
P6—Cu3—P4—C40	−5.1 (2)	C46—C41—C42—C43	0.0
Br3—Cu3—P4—C40	174.09 (19)	P4—C41—C42—C43	−178.5 (3)
P6—Cu3—P4—C41	−129.00 (15)	C41—C42—C43—C44	0.0
Br3—Cu3—P4—C41	50.18 (16)	C42—C43—C44—C45	0.0
P6—Cu3—P4—C47	113.66 (14)	C43—C44—C45—C46	0.0
Br3—Cu3—P4—C47	−67.17 (14)	C44—C45—C46—C41	0.0
P2—Cu2—P5—C73	−62.1 (2)	C42—C41—C46—C45	0.0
Br1—Cu2—P5—C73	169.9 (2)	P4—C41—C46—C45	178.5 (3)
Br2—Cu2—P5—C73	64.2 (2)	C40—P4—C47—C48	11.7 (3)
P2—Cu2—P5—C67	62.10 (17)	C41—P4—C47—C48	119.8 (2)
Br1—Cu2—P5—C67	−65.86 (16)	Cu3—P4—C47—C48	−117.0 (2)
Br2—Cu2—P5—C67	−171.53 (15)	C40—P4—C47—C52	−169.3 (2)
P2—Cu2—P5—C66	178.3 (2)	C41—P4—C47—C52	−61.2 (2)
Br1—Cu2—P5—C66	50.4 (2)	Cu3—P4—C47—C52	61.9 (2)
Br2—Cu2—P5—C66	−55.3 (2)	C52—C47—C48—C49	0.0
P4—Cu3—P6—C65	−35.5 (2)	P4—C47—C48—C49	178.9 (3)
Br3—Cu3—P6—C65	145.41 (19)	C47—C48—C49—C50	0.0
P4—Cu3—P6—C59	−156.70 (18)	C48—C49—C50—C51	0.0
Br3—Cu3—P6—C59	24.17 (19)	C49—C50—C51—C52	0.0
P4—Cu3—P6—C53	80.54 (16)	C50—C51—C52—C47	0.0
Br3—Cu3—P6—C53	−98.59 (16)	C48—C47—C52—C51	0.0
C7—P1—C1—C2	−21.3 (3)	P4—C47—C52—C51	−179.0 (3)
C13—P1—C1—C2	−128.6 (3)	C65—P6—C53—C54	−26.2 (3)
Cu1—P1—C1—C2	109.2 (2)	C59—P6—C53—C54	80.5 (3)
C7—P1—C1—C6	162.7 (3)	Cu3—P6—C53—C54	−149.8 (2)
C13—P1—C1—C6	55.3 (3)	C65—P6—C53—C58	152.9 (3)
Cu1—P1—C1—C6	−66.8 (3)	C59—P6—C53—C58	−100.5 (3)
C6—C1—C2—C3	0.0	Cu3—P6—C53—C58	29.2 (3)
P1—C1—C2—C3	−176.0 (3)	C58—C53—C54—C55	0.0
C1—C2—C3—C4	0.0	P6—C53—C54—C55	179.0 (3)
C2—C3—C4—C5	0.0	C53—C54—C55—C56	0.0
C3—C4—C5—C6	0.0	C54—C55—C56—C57	0.0
C4—C5—C6—C1	0.0	C55—C56—C57—C58	0.0
C2—C1—C6—C5	0.0	C56—C57—C58—C53	0.0
P1—C1—C6—C5	176.1 (3)	C54—C53—C58—C57	0.0
C1—P1—C7—C8	109.2 (3)	P6—C53—C58—C57	−179.0 (3)
C13—P1—C7—C8	−145.1 (3)	C65—P6—C59—C60	−127.1 (3)
Cu1—P1—C7—C8	−17.3 (3)	C53—P6—C59—C60	125.8 (3)
C1—P1—C7—C12	−70.1 (3)	Cu3—P6—C59—C60	0.7 (3)
C13—P1—C7—C12	35.7 (3)	C65—P6—C59—C64	50.8 (3)
Cu1—P1—C7—C12	163.46 (19)	C53—P6—C59—C64	−56.2 (3)
C12—C7—C8—C9	0.0	Cu3—P6—C59—C64	178.7 (2)
P1—C7—C8—C9	−179.2 (3)	C64—C59—C60—C61	0.0
C7—C8—C9—C10	0.0	P6—C59—C60—C61	178.0 (3)
C8—C9—C10—C11	0.0	C59—C60—C61—C62	0.0
C9—C10—C11—C12	0.0	C60—C61—C62—C63	0.0
C10—C11—C12—C7	0.0	C61—C62—C63—C64	0.0
C8—C7—C12—C11	0.0	C62—C63—C64—C59	0.0
P1—C7—C12—C11	179.2 (3)	C60—C59—C64—C63	0.0
C7—P1—C13—C14	63.0 (4)	P6—C59—C64—C63	−177.9 (4)
C1—P1—C13—C14	170.4 (4)	C59—P6—C65—C66	72.2 (4)
Cu1—P1—C13—C14	−68.2 (4)	C53—P6—C65—C66	−178.1 (4)
P1—C13—C14—P2	107.0 (4)	Cu3—P6—C65—C66	−57.5 (4)
C21—P2—C14—C13	62.5 (4)	P6—C65—C66—P5	−166.1 (3)
C15—P2—C14—C13	168.8 (3)	C73—P5—C66—C65	178.6 (4)
Cu2—P2—C14—C13	−62.7 (4)	C67—P5—C66—C65	68.6 (4)
C21—P2—C15—C16	118.2 (3)	Cu2—P5—C66—C65	−56.0 (4)
C14—P2—C15—C16	10.8 (3)	C73—P5—C67—C68	136.8 (3)
Cu2—P2—C15—C16	−117.7 (2)	C66—P5—C67—C68	−115.5 (3)
C21—P2—C15—C20	−64.1 (3)	Cu2—P5—C67—C68	8.6 (3)
C14—P2—C15—C20	−171.5 (3)	C73—P5—C67—C72	−48.7 (3)
Cu2—P2—C15—C20	60.1 (3)	C66—P5—C67—C72	59.0 (3)
C20—C15—C16—C17	0.0	Cu2—P5—C67—C72	−177.0 (2)
P2—C15—C16—C17	177.7 (3)	C72—C67—C68—C69	0.0
C15—C16—C17—C18	0.0	P5—C67—C68—C69	174.6 (3)
C16—C17—C18—C19	0.0	C67—C68—C69—C70	0.0
C17—C18—C19—C20	0.0	C68—C69—C70—C71	0.0
C18—C19—C20—C15	0.0	C69—C70—C71—C72	0.0
C16—C15—C20—C19	0.0	C70—C71—C72—C67	0.0
P2—C15—C20—C19	−177.8 (3)	C68—C67—C72—C71	0.0
C14—P2—C21—C22	36.5 (3)	P5—C67—C72—C71	−174.3 (3)
C15—P2—C21—C22	−71.0 (3)	C67—P5—C73—C74	−15.8 (6)
Cu2—P2—C21—C22	163.24 (17)	C66—P5—C73—C74	−121.2 (5)
C14—P2—C21—C26	−146.6 (2)	Cu2—P5—C73—C74	113.2 (5)
C15—P2—C21—C26	106.0 (2)	C67—P5—C73—C78	170.8 (4)
Cu2—P2—C21—C26	−19.9 (2)	C66—P5—C73—C78	65.3 (5)
C26—C21—C22—C23	0.0	Cu2—P5—C73—C78	−60.3 (5)
P2—C21—C22—C23	176.8 (3)	C78—C73—C74—C75	−1.0 (9)
C21—C22—C23—C24	0.0	P5—C73—C74—C75	−174.4 (5)
C22—C23—C24—C25	0.0	C73—C74—C75—C76	1.4 (12)
C23—C24—C25—C26	0.0	C74—C75—C76—C77	−0.2 (13)
C24—C25—C26—C21	0.0	C75—C76—C77—C78	−1.4 (12)
C22—C21—C26—C25	0.0	C74—C73—C78—C77	−0.5 (9)
P2—C21—C26—C25	−177.0 (3)	P5—C73—C78—C77	173.4 (5)
C33—P3—C27—C28	143.4 (2)	C76—C77—C78—C73	1.8 (11)
C39—P3—C27—C28	−109.2 (3)		

### Hydrogen-bond geometry (Å, °)

**Table:**

*D*—H···*A*	*D*—H	H···*A*	*D*···*A*	*D*—H···*A*
C24—H24···Br1^i^	0.93	2.92	3.560 (3)	127
C65—H65A···Br1	0.97	2.85	3.576 (5)	132
C40—H40A···Br2	0.97	2.86	3.675 (5)	142

Symmetry codes: (i) *x*−1/2, −*y*+3/2, *z*.
